# Outbreak of Crimean-Congo haemorrhagic fever with atypical clinical presentation in the Karak District of Khyber Pakhtunkhwa, Pakistan

**DOI:** 10.1186/s40249-018-0499-z

**Published:** 2018-11-19

**Authors:** Khalid Rehman, Muhammad Asif Khan Bettani, Luzia Veletzky, Shaheen Afridi, Michael Ramharter

**Affiliations:** 1 Department of Medicine I, Division of Infectious Diseases and Tropical Medicine, Medical University of Vienna, Währinger Gürtel 18-20, 1090 Vienna, Austria; 2 Department of health Khyber Pakhtunkhwa, Gate # 5 opposite Pearl Continental hotel Main GT road Peshawar, Peshawar, 25000 Pakistan; 3 Institute of Tropical Medicine, University of Tübingen, Wilhelmstraße 27, 72074 Tübingen, Germany; 4 Department of Tropical Medicine, Bernhard Nocht Institute for Tropical Medicine & I Department of Medicine, University Medical Center Hamburg-Eppendorf, Bernhard-Nocht-Straße 74, 20359 Hamburg, Germany

**Keywords:** Crimean Congo haemorrhagic fever, GI symptoms, Outbreak, Contact tracing

## Abstract

**Background:**

Crimean-Congo haemorrhagic fever (CCHF) is a potentially fatal disease endemic in Pakistan. The causative virus is transmitted by the bite of *Hyalomma* ticks or by contact with infected blood or tissue. First cases of the disease were reported in Pakistan in 1976 but regular outbreaks have been observed since the year 2000. A huge agricultural base with more than 175 million livestock, the concomitant presence of *Hyalomma* ticks and a lack of precautionary measures to prevent transmission lead to a considerable risk for exposed populations to contract CCHF in Pakistan. At the same time, secondary cases contracted by nosocomial transmission are reported from hospitals.

**Case presentation:**

Here we present an outbreak of CCHF with four of six patients succumbing to the disease before the suspicion for CCHF was raised. Importantly, the main clinical features of these cases were gastrointestinal symptoms without any clinical signs of bleeding. Only the last two patients in this outbreak presented with typical signs of bleeding disorder and were then confirmed being infected by CCHF. Confirmation of diagnosis was done at the National Institute of Health by real-time RT-PCR.

**Conclusions:**

This case series highlights the importance of early clinical suspicion for CCHF in exposed individuals and the need for improved precautionary measures against the spread of CCHF within the Pakistani population and hospitals.

**Electronic supplementary material:**

The online version of this article (10.1186/s40249-018-0499-z) contains supplementary material, which is available to authorized users.

## Multilingual abstract

Please see Additional file 1 for translations of the abstract into the five official working languages of the United Nations.

## Background

Crimean-Congo haemorrhagic fever (CCHF) is a potentially deadly disease with mortality of up to 70% [1, 2]. The disease was first described in 1944 in Crimea where it affected more than 200 individuals and lead to severe bleeding as clinical hallmark of the disease [3]. The causative virus (*Nairovirus*) belongs to the Bunyaviridae family and was first isolated in Congo in 1956 [4, 5]. The virus displays a zoonotic life cycle with animal to animal transmission and is transmitted to humans either by exposure to the bite of *Hyalomma* ticks or by direct contact with infected blood and tissue [6, 7].

CCHF is endemic in Pakistan, where the climatic conditions are suitable for the transmitting ticks, hence allowing the spread of the virus [8–10]. Within the country agriculture and farming form the base of socioeconomic structures and as counted by the 2015 national economic survey performed by the Government of Pakistan more than 175 million farm animals are held within the country [11]. These huge numbers of animals form the epidemiological basis for the viral reservoir and for virus transmission. Further, migration of endemic populations from higher prevalence countries such as Afghanistan has been described as one important factor of disease dissemination in Pakistan. Additionally, it has been reported that traditional nomadic life styles, which exist in Afghanistan as well as in Pakistan, favour CCHF infections [12, 13]. The first case of CCHF in Pakistan was reported in January 1976 [14]. Since then only a few cases have been reported from diverse regions within the country [15–18]. However, in recent years case numbers have constantly risen and only within the period from 2012 to 2014 a total of 196 cases and 48 deaths have been reported [19]. Yet, an ongoing increase in death numbers was seen in 2016 with 20 reported fatalities [20].

The clinical presentation of CCHF can range from self-limiting flu-like to life threating symptoms [21, 22]. If the disease progresses it presents in four stages i.e. incubation, pre-haemorrhagic, haemorrhagic, and convalescence stage. The incubation period can range from 1 to 3 days following a bite by an infected tick and 5–6 days following exposure to contaminated tissue [23]. During the pre-haemorrhagic stage, the presenting signs and symptoms include fever, headache, myalgia, abdominal pain, photophobia and sometimes, gastrointestinal symptoms [15, 24–27]. The consecutive stage is haemorrhagic and patients suffer from epistaxis, hematemesis, melena, gum bleeding, petechial bleeding, haematuria and other haemorrhagic signs [16].

### Outbreak alert

Karak is a southern district of Khyber Pakhtunkhwa (KPK) Province. In May 2017, a cluster of four deaths was reported from Ambiri Village in the district of Karak. In total six patients had presented with severe diarrhoea, nausea and vomiting. Initially, this was suspected to be an outbreak of a yet undiagnosed gastrointestinal disease. Two of the patients later also developed signs of severe bleeding disorder. Within four days, four out of the six patients had died. Based on the rapid evolvement of disease, fatalities in apparently healthy individuals and the unknown cause of death an investigation of the outbreak was started. The investigating team collected all available information and health data, which are presented below. The case definitions used during the investigations are shown in Table 1.

**Table 1:** Case definitions used during the outbreak investigation

1.	Suspected case	Patient with sudden onset of illness and fever for more than 3 days and less than 10 days in Crimean-Congo haemorrhagic fever (CCHF) endemic area or those among contact with livestock.
2.	Probable case	Suspected case with thrombocytopenia and any two of the following: petechial or purpuric rash, epistaxis, hematemesis, haemoptysis, blood in stools, ecchymosis, gum bleeding, other haemorrhagic symptom.
3.	Confirmed case	CCHF confirmed from NIH through ELISA/PCR

*NIH* National Institute of Health, *ELISA* enzyme linked immunosorbent assay, *PCR* polymerase chain reaction

## Case presentations

### Case 1

On 26th May, 2017, a 49 years old male patient, from Ambiri Village demonstrated predominant gastrointestinal symptoms of nausea and diarrhoeas accompanied by fever. He was treated at home by paramedics but his condition deteriorated and he died suddenly after experiencing heavy diarrhoeal episodes. Since he was not taken to a hospital, further investigations could not be done. According to his family he has been involved in the slaughter of a cow before the onset of symptoms.

### Cases 2 and 3

On 28th May, 2017, another male patient, 40 years old, from the same village and neighbour of the first case, developed diarrhoea accompanied by excessive nausea and vomiting and was therefore referred to a local hospital. He was admitted and routine investigations were performed. The investigating team did however not get access to the results of these laboratory investigations. According to the treating physician and the attendants of the patient, no clinical signs of bleeding were observed. In the meantime, the patient’s 15 years old son similarly developed progressive vomiting and diarrhoea and was taken to the hospital. Symptoms were still considered to be due to common gastroenteritis and hence both patients received rehydration therapy. As the clinical state of the patient worsened further, case 2 was referred to a tertiary care hospital in Peshawar. He did not survive the transfer and the corpse was then transported back to the village for funeral rituals. His son, who was case 3 in this investigation, was sent home, despite of his bad state, to attend last rituals of the funeral of his father. There he similarly died within the following days.

### Case 4

On 29th May, 2017, a fourth person developed gastrointestinal symptoms and was initially treated by a local physician for gastroenteritis and dehydration in Karak. Due to his worsening clinical condition, he was referred to the Lady Reading Hospital Peshawar. There, he was transferred from the emergency unit to the gastroenterology department. Before further investigations were done, the patient died on 31st May.

### Case 5

**Fig. 1:**
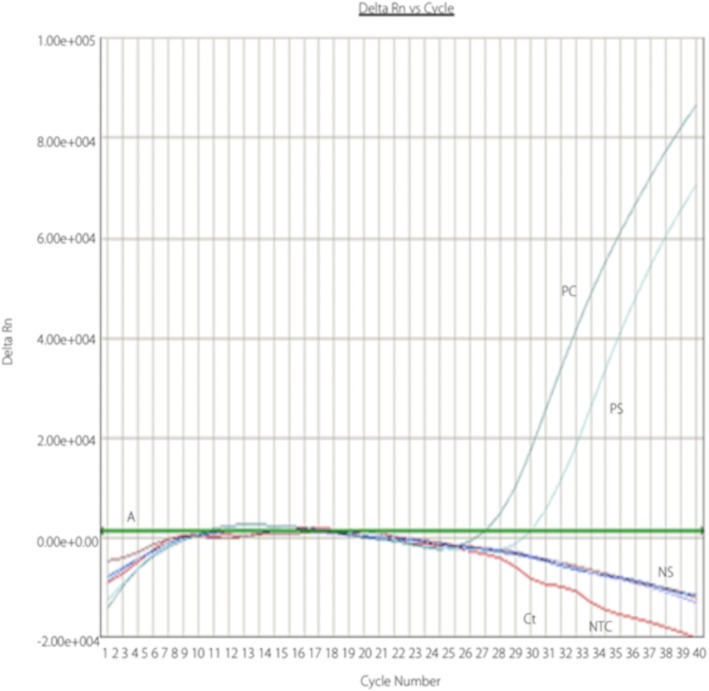
Depiction of a positive real-time RT-PCR result for CCHF virus detection. Amplification plots generated during RT-PCR for virus detection are shown. The number of PCR cycles is given on X-axis and the Delta Rn values are plotted on Y-axis. The green line is called the threshold baseline (A). Delta Rn represents the change in the fluorescent cycle. The fractional cycle number at which that sample crosses the threshold is called the cycle of threshold (=Ct). As shown in the graph the fluorescent peak of positive samples (=PS) crosses the threshold baseline while the negative samples (=NS) yield very low fluorescent signals below the threshold baseline

The fifth case was a 53 years old male and had been involved in butchering and skinning of the same cow, as the other patients. He developed similar gastrointestinal symptoms as well as additional haemorrhagic signs (melena) and also was admitted to Lady Reading Hospital Peshawar. His blood specimen was sent to the hospitals laboratory for routine investigations and additionally to the National Institute of Health (NIH) for detection of the Dengue NS-1 antigen, CCHF virus antigen and genomic RNA, using a real-time reverse transcriptase polymerase chain reaction (RT-PCR) (Fig. 1) assay [28]. The CCHF-PCR showed a positive result confirming the suspected infection. The patient was treated with ribavirin and supportive treatment and survived the infection.

### Case 6

The sixth case was a male adult, who was a relative of case 5 and had also been involved in slaughtering and skinning of the cow. After suffering from unspecific clinical symptoms, he developed haemorrhagic fever and was taken to a hospital in the capital city Islamabad. He was also tested for CCHF and was confirmed by the NIH to be positive as well (personal communication). Further details about his treatment could not be obtained, but he did survive the acute episode of disease.

### Outbreak investigation

The outbreak investigation revealed that a terminally ill cow had been slaughtered in the village of the patients and that the meat had been distributed to all the 110 households of the village. The gastrointestinal symptoms of the cases were the main presenting feature so the initial hypothesis had been that the illness might be associated with the ingestion of the cow meat. But the investigation team determined that approximately more than 300 people had eaten some of the meat but only six had developed symptoms. All of these six patients had also been involved in slaughtering of the cow, handling the carcass and skinning of the animal. Even though great efforts were made to retrospectively obtain samples from all of the presented patients, PCR diagnosis of CCHF virus infection was done in just two of the cases. Diagnosis of the other four patients was based on clinical symptoms, history of patients and close link to the confirmed cases. The predominant symptoms in the presented cases were of gastrointestinal type.

### Contact tracing

The investigating team visited the village and it was found that in total there were 21 contacts who had been exposed to a high risk of contracting CCHF by handling the infected cow. This included family members and relatives of the patients, health workers involved in patient care, a veterinary doctor who had had contact with the cow, as well as those involved in the slaughter and skinning of the cow. Blood was taken from all the 21 contacts and sent to NIH for CCHF testing. All of the contacts results turned out negative.

## Discussion and conclusions

Within this case report several of the challenges are presented that health care personal has to face when confronted with CCHF in Pakistan. These include problems with clinical suspicion and availability of diagnostic services for CCHF. At present only two facilities - NIH in Islamabad and the Agha Khan Hospital in Karachi - are capable of diagnosing CCHF. However, sending specimens to these facilities from peripheral hospitals is costly and the lack of monetary resources can constitute an important barrier in adequate laboratory assessment. Secondly, results will take several days until being available. This poses an important operational challenge as the patient may have already have succumbed to the disease and further transmission of the virus may have occurred either in the health facility or within the family of the patient.

Over the past decades CCHF has been reported only sporadically in different parts of the country and almost all previously reported CCHF cases in Pakistan presenting to hospitals were characterized by haemorrhagic symptoms [15–18, 29, 30]. Due to the atypical presentation in this case series, CCHF has not been among the differential diagnosis of the treating physicians. Similar to this outbreak, CCHF is often only considered in a late stage of disease, when the typical haemorrhagic manifestations start to appear. This delay in confirmation of CCHF may lead to inadequate patient management, including a lack of protective measures for health care personal and family members. This, again increases the risk for secondary infections within the hospital and the community.

Diarrheal diseases are highly prevalent in Pakistan during the summer seasons – a period with increased risk for contracting CCHF due to suitable climatic conditions for the transmitting ticks. As diarrhoea can be an early clinical sign of CCHF, it is important for health care workers in CCHF endemic areas to maintain a high level of suspicion. To take a rigorous history of contact and exposure to animals, travel details and profession of patients presenting with diarrheal diseases is an important step when establishing differential diagnoses. If the history points to a possible infection with CCHF, the disease should be ruled out immediately. Considering CCHF as differential diagnosis early in the clinical course is also paramount for use of adequate personal protective measures, which are necessary to prevent nosocomial transmission of the disease [31–34]. To avoid future outbreaks and overcome the described challenges, awareness of the disease should be raised in health care personnel, including areas where CCHF has not been described before. Additionally, clinicians should be informed about the likelihood of atypical presentations in CCHF. Concomitantly, specimen transfer and diagnostics need to be facilitated and the use of protective measures should be enforced as long as diagnosis is unclear. Similarly, the communities which are at risk of contracting CCHF should be sensitized about the disease, its transmission and prevention. This can be done through mass campaigns using electronic, print and social media. The campaigns should not be limited only to the Eidul Azha season but should be continued throughout the year in the high-risk areas.

Currently there is no surveillance system in place to report the disease early, especially in Baluchistan, an underdeveloped region of Pakistan. In Khyber Pakhtunkhwa Province, however, a new initiative has recently been launched with the name of Integrated Disease Surveillance and Response System (IDSRS) [35]. The main aim of IDSRS is early detection of any epidemic, rapid and appropriate response.

Clearly this case series has several limitations, including a lack of testing of biological samples and specific details or laboratory results or history of the patients. Conversely, this is also an appropriate reflection of real life settings in local hospitals in rural Pakistan and particularly in resource limited regions of Western Pakistan. It underlines the importance of early clinical suspicion of this rare but dangerous disease when diagnostic possibilities are far off and doctors may have to rely on basic examination and clinical experience. It also presents how traditional habits, such as unprotected slaughtering of cows in groups or religious ceremonies influence disease dissemination and outcome.

In summary, this outbreak reminds us that CCHF may present with rather unspecific symptoms and can have a rapidly fatal course of disease. Therefore, awareness of the disease has to be raised. Trainings for health workers focussing on diagnosis and treatment but also on notifying health authorities about new cases should become a priority in these affected regions.

## Additional file

Additional file 1: Multilingual abstracts in the five official working languages of the United Nations. (PDF 718 kb)

## Abbreviations

CCHF: Crimean-congo haemorrhagic fever
ELISA: Enzyme linked immunosorbent assay
IDSRS: Integrated disease surveillance and response system
KPK: Khyber pakhtunkhwa province
NIH: National institute of health
RT-PCR: Reverse transcriptase polymerase chain reaction
