# Supplementary material for: Outbreak of Crimean-Congo haemorrhagic fever with atypical clinical presentation in the Karak District of Khyber Pakhtunkhwa, Pakistan
Source: Infect Dis Poverty. 2018 Nov 19;7:116. doi: 10.1186/s40249-018-0499-z (PMC6240963; doi:10.1186/s40249-018-0499-z)
Supplement: Supplementary file 1 — Multilingual abstracts in the five official working languages of the United Nations. (PDF 718 kb) [file 40249_2018_499_MOESM1_ESM.pdf]

Translation of the abstract into the five official working languages of the United Nations

تفشي حمى القرم والكونغو النزفية مع أعراض سريرية غير عادية في مقاطعة كاراك من اقليم خيبر باختونخوا، باكستان.

خالد رحمان، ومحمد أصف خان بيتاني، ولوزيا فيليتسكي، وشاهين أفريدي و مايكل رامهارتَر.

مُلَخَّص

خلفية: حمى القرم والكونغو النزفية هي أحد الأمراض المميتة المتوطنة في باكستان. ينتقل الفيروس المسبب لهذه الحمى عن طريق لسعة قراد الزُّجَاجِيُّ العَيْن أو عن طريق الاتصال بدم أو أنسجة ملوثة. رصدت أول الحالات لهذه الحمى في باكستان عام 1976 ولكن لوحظ التفشي المتكرر لهذه الحمى منذ عام 2000. إن القاعدة الزراعية الضخمة مع مايزيد عن 175 مليون رأس من الماشية، ووجود القراد زجاجي العين بالإضافة إلى نقص التدابير الوقائية لمنع الانتقال، كل هذه الأمور تؤدي إلى خطر محتمل من تعرض السكان في باكستان لحمى القرم والكونغو النزفية. وفي نفس الوقت، رصدت المستشفيات حالات من الدرجة الثانية أصيبت عن طريق عدوى المستشفيات.

عرض الحالة: نعرض هنا فترة تفشت فيها حمى القرم والكونغو النزفية مع استسلام أربعة من كل ستة مرضى لهذا المرض قبل الاشتباه حتى بحمى القرم والكونغو النزفية. ومن المهم ذكر أن من أهم المظاهر السريرية لهذه الحالات هي أعراض معوية بدون أي علامات سريرية تدل على وجود نزيف. أظهر فقط آخر اثنين من المرضى خلال فترة تفشي المرض علاماتٍ نموذجية لاضطرابات نزفية وتم بعدها تأكيد إصابتهما بحمى القرم والكونغو النزفية. كما استُخدم نظام PCR-RT لتأكيد التشخيصات في المعهد الوطني للصحة في نفس الوقت.

الخاتمة:تؤكد سلسلة الحالات هذه على أهمية الاشتباه المبكر لحمى القرم والكونغو النزفية لدى الأفراد المعرضين لها والحاجة إلى تدابير وقائية محسنة ضد انتشار هذه الحمى بين سكان باكستان ومستشفياتها.

Translated from English version into Arabic by Ghada Alluhaidan, proofread by Jamal Alsamarrai, through

TRANSLATORS WITHOUT BORDERS

巴基斯坦开伯尔-普赫图赫瓦省卡拉克地区暴发非典型临床表现克里米亚-刚果出血热

Khalid Rehman, Muhammad Asif Khan Bettani, Luzia Veletzky, Shaheen Afridi, Michael Ramharter

摘要

摘要

引言：克里米亚-刚果出血热（CCHF）是流行于巴基斯坦的一种致命疾病，致病病毒通过璃眼蜱叮咬或接触感染者的血液、组织进行传播。巴基斯坦于 1976 年报告首例病例，但 2000 年以来观察到规律性暴发。巴基斯坦是一个拥有超过 1.75 亿牲畜的巨大农业基地。在巴基斯坦，璃眼蜱与人类相伴共存，且缺乏阻断传播的预防措施，导致暴露人群感染 CCHF 的风险很高。与此同时，医院也报告了由院内感染导致的续发病例。

病例报告：我们报告一个 CCHF 暴发疫情，6 名患者中有 4 人死于该病，却未被怀疑是由 CCHF 引起的。因这 4 例病例的主要临床特征是胃肠道症状，未见任何出血的临床症状。其余

两个病例出现了典型的出血障碍症状，被确诊为 CCHF 感染。本次疫情的病例诊断是国立卫生研究院通过实时荧光定量 PCR 方法证实的。
结论：本次 CCHF 系列病例强调在临床早期应高度怀疑暴露个体感染 CCHF，且需要改进预防措施以防止 CCHF 在巴基斯坦人群和医院内传播。

Translated from English version into Chinese by Sheng-Lin Wang, edited by Pin Yang

Éclosion de fièvre hémorragique de Crimée-Congo avec présentation clinique atypique dans le district de Karak de Khyber Pakhtunkhwa, au Pakistan

Khalid Rehman, Muhammad Asif Khan Bettani, Luzia Veletzky, Shaheen Afridi, Michael Ramharter

Résumé

Contexte: La fièvre hémorragique de Crimée-Congo (FHCC) est une maladie potentiellement mortelle, endémique au Pakistan. Le virus responsable est transmis par la morsure de tiques de type *Hyalomma* ou en entrant en contact avec du sang ou des tissus infectés. Les premiers cas de la maladie ont été signalés au Pakistan en 1976, mais des éclosions régulières sont observées depuis l'an 2000. Une structure agricole considérable de plus de 175 millions de têtes de bétail, la présence concomitante de tiques *Hyalomma* et le manque de mesures préventives contre la transmission entraînent un risque considérable pour les populations exposées de contracter la FHCC au Pakistan. Dans le même temps, des cas secondaires contractés par transmission nosocomiale sont signalés par les hôpitaux.
Présentation de cas: Nous présentons ici une éclosion de FHCC, avec quatre patients sur six succombant à la maladie avant que la suspicion de FHCC ne soit soulevée. Fait important, les principales caractéristiques cliniques de ces cas étaient des symptômes gastro-intestinaux sans aucun signe clinique de saignement. Seuls les deux derniers patients de cette éclosion présentaient des signes typiques de troubles de la coagulation et ont ensuite été confirmés infectés par la FHCC. La confirmation des diagnostics a été effectuée à l'Institut national de la santé par RT-PCR en temps réel.
Conclusions: Cette série de cas souligne l'importance de la suspicion clinique précoce de la FHCC chez les individus exposés et la nécessité d'améliorer les mesures de précaution contre la propagation de la FHCC au sein de la population et des hôpitaux pakistanais.

Translated from English version into French by Iris Soliman, proofread by Maëva P, through

Вспышка Конго-крымской геморрагической лихорадки с нетипичным клиническим проявлением в Каракском районе провинции Хайбер-Пахтунхва, Пакистан

Халид Рехман, Мухаммад Асиф Хан Беттани, Люсия Велецкий, Шахин Африди, Майкл Рамхартер

Аннотация

Справочная информация: Конго-крымская геморрагическая лихорадка (ККГЛ) является потенциально смертельной болезнью, эндемичной в Пакистане. Вирус-возбудитель инфекции передается путем укуса клещей *Гиалома* или при контакте с инфицированной кровью или тканью. Первые случаи заболевания были зарегистрированы в Пакистане в 1976 году, однако с 2000 года наблюдались регулярные вспышки. В огромной сельскохозяйственной базе с более 175 миллионами скота, сопутствующее присутствие клеща *Гиаломы,* а также отсутствие профилактических мер приводят к значительному риску заражения среди подверженных воздействию ККГЛ групп населения в Пакистане. В то же время из больниц поступают сведения о появлении вторичных случаев, связанных с нозокомиальной передачей.

Представление случая: В данном случае рассматривается вспышка ККГЛ у четырех из шести пациентов, которые стали жертвами болезни до того, как возникло подозрение о заражении ККГЛ. Важно отметить, что основными клиническими особенностями этих случаев были желудочно-кишечные симптомы без каких-либо клинических признаков кровотечения. Только у последних двух пациентов в этой вспышке были типичные признаки свертываемости крови, и затем было подтверждено, что они инфицированы ККГЛ. Подтверждение диагноза проводилось в Национальном институте здравоохранения по ОТ-ПЦР в режиме реального времени.

Выводы: В этой серии случаев подчеркивается важность раннего клинического предположения появления ККГЛ у подверженных воздействию лиц, а также необходимость совершенствования профилактических мер по борьбе с распространением ККГЛ среди населения Пакистана и в больницах.

Translated from English version into Russian by Milina Nikolic, proofread by Liudmila Tomanek, through

TRANSLATORS
WITHOUT BORDERS

Brote de fiebre hemorrágica de Crimea-Congo con presentación clínica atípica en el distrito de Karak, provincia de Khyber Pakhtunkhwa (Pakistán)

Autores: Khalid Rehman, Muhammad Asif Khan Bettani, Luzia Veletzky, Shaheen Afridi, Michael Ramharter

Resumen

Contexto: La fiebre hemorrágica de Crimea-Congo (FHCC) es una enfermedad endémica de Pakistán que puede resultar letal. El virus causante de la enfermedad se transmite por la picadura de la garrapata *Hyalomma* o por contacto con la sangre o el tejido infectado. Los primeros de casos de esta enfermedad

se detectaron en 1976, aunque se han ido observando brotes periódicos desde 2002. La economía agrícola de la zona, con más de 175 cabezas de ganado, junto con la presencia de la garrapata *Hyalomma* y la falta de medidas preventivas dan lugar a que la población esté expuesta a un riesgo considerable de contraer la enfermedad. Asimismo, los hospitales han detectado casos en los que la enfermedad se ha contraído por infección nosocomial.

Presentación del caso: Presentamos un brote de fiebre hemorrágica de Crimea-Congo en el que cuatro de seis pacientes murieron a causa de dicha enfermedad antes de que se les diagnosticara. Cabe destacar que las principales características clínicas de dichos casos fueron síntomas gastrointestinales sin signos clínicos de hemorragia. Sólo los dos últimos pacientes presentaron signos típicos de trastorno hemorrágico, momento en que se confirmó que estaban infectados por la enfermedad. La confirmación del diagnóstico se realizó en el Instituto Nacional de Salud a través del método de reacción en cadena de la polimerasa con transcriptasa inversa.

Conclusiones: Esta serie de casos pone de manifiesto la importancia del diagnóstico clínico temprano de la fiebre hemorrágica de Crimea-Congo en individuos expuestos al mismo, así como la necesidad de medidas preventivas más efectivas contra la propagación de la enfermedad entre la población y en los hospitales paquistaníes.

Translated from English version into Spanish by Marta Callava Linares, proofread by Angelines G, through

TRANSLATORS
WITHOUT BORDERS
